# Inherited thrombotic thrombocytopenic purpura mimicking immune thrombocytopenic purpura during pregnancy: a case report

**DOI:** 10.1186/s13256-017-1545-3

**Published:** 2018-01-22

**Authors:** Valter Romão de Souza, Ana Beatriz Cavalcante de Oliveira, Ana Maria Vanderlei, Amanda Queiroz da Mota Silveira Aroucha, Bruna Pontes Duarte, Aureli Nunes Machado, Lívia Netto Chaer, Cláudia Wanderley de Barros Correia, Maria da Conceição de Barros Correia, Manuela Freire Hazin Costa

**Affiliations:** 1 Department of Internal Medicine, Federal University of Pernambuco, Haematology, Av. Prof. Moraes Rego 1235, 50670-90 Recife, Brazil; 2 Liga Acadêmica de Hematologia da Universidade Federal de Pernambuco, Av. Prof. Moraes Rego 1235, 50670-901 Recife, Brazil; 3 Fundação de Hematologia e Hemoterapia de Pernambuco (HEMOPE), R. Joaquim Nabuco 171, 52011-000 Recife, Brazil; 4 Instituto de Medicina Integral Prof. Fernando Figueira (IMIP), R. dos Coelhos 300, 50070-550 Recife, Brazil; 5 Hospital das Clínicas, Federal University of Pernambuco, Av. Prof. Moraes Rego 1235, 50670-90 Recife, Pernambuco Brazil

**Keywords:** ADAMTS13, Congenital thrombotic thrombocytopenic purpura, Upshaw–Schulman syndrome, Pregnancy

## Abstract

**Background:**

Thrombotic thrombocytopenic purpura is a very rare hereditary blood deficiency disorder of ADAMTS13 (von Willebrand factor-cleaving protease) and a life-threatening thrombotic microangiopathy characterized by thrombocytopenia and microangiopathic hemolytic anemia. The deficiency in ADAMTS13 metalloprotease, which cleaves the von Willebrand factor, may be congenital or acquired. The congenital form is caused by inherited mutations in the *ADAMTS13* gene. The diagnosis is challenging due to the nonspecific signs and symptoms, as well as the rarity of the disease.

**Case presentation:**

We present an unusual case of a 20-year-old feoderm woman from northeast region of Brazil who manifested thrombocytopenia during her pregnancy which was believed to be immune thrombocytopenic purpura.

**Conclusions:**

Considering the importance of a differential diagnosis of thrombotic microangiopathic disorders, congenital thrombotic thrombocytopenic purpura may mimic the signs and symptoms of pre-eclampsia/eclampsia, hemolysis with elevated liver enzymes and low platelet count syndrome, and atypical hemolytic-uremic syndrome. It should be considered in suspect cases in patients with an ADAMTS13 activity at 5% without ADAMTS13 antibodies.

## Background

Thrombotic thrombocytopenic purpura (TTP) is a serious condition resulting from platelet aggregation mainly at a microcirculation level. It results in thrombocytopenia, microangiopathic hemolytic anemia (MAHA), and occlusive ischemia [1]. The occlusion is caused by widespread microthrombi composed basically of platelets and von Willebrand factor (vWF). The target organ of this occlusive ischemia process is mainly the brain. The kidneys and the gastrointestinal tract are less often affected. During the disease, protein deficiency promotes the formation of abnormally large von-Willebrand multimers, attracting platelets and fibrin anchors and creating a widespread prothrombotic feedback process with a systemic deposition of platelet thrombi on capillary vessels and arteries. The disease usually results from a reduction in the activity of the enzyme ADAMTS13, a metalloprotease responsible for vWF cleavage [2]. This reduction is due to the presence of anti-ADAMTS13 autoantibodies (acquired TTP) or *ADAMTS13* gene mutation (inherited TTP).

Acquired TTP is a rare thrombotic microangiopathy with an incidence of approximately 2.2 cases per million per year [3]. Congenital or inherited TTP represents less than 5% of all cases of TTP and the annual incidence is estimated at less than 1/1,000,000 [4]. Neonates and children usually manifest congenital TTP. However, females in certain situations such as pregnancy may present acute TTP attacks [4]. Females are more frequently affected than males, with almost twice the incidence, because of the risk of an acute TTP attack precipitated during pregnancy [5, 6]. The symptomatology is strongly represented by neurological changes, usually with a sudden appearance, and marked by a picture of recurrent relapses that signal the persistence of the disease during the course of treatment. In addition, the patient may have kidney manifestations and fever.

With regard to the importance of a differential diagnosis of thrombotic microangiopathic disorders, we present a case of inherited ADAMTS13 deficiency with initial clinical features that mimicked a recurrent immune thrombocytopenic purpura (ITP). Because inherited TTP is extremely rare and complicates the course of pregnancy, the differential diagnosis of this disease is important. For this reason, diagnosis of microangiopathic disorders should be based on clinical symptoms and laboratory findings.

## Case presentation

A 20-year-old feoderm woman from the Northeastern region of Brazil was hospitalized during her first pregnancy in the Fundação de Hematologia e Hemoterapia de Pernambuco (HEMOPE), a hematologic reference hospital. She presented microcytosis (mean corpuscular volume of 74 fL/red cell), hypochromic anemia (hemoglobin of 6.5 g/dL and mean corpuscular hemoglobin of 21.8 g/dL), thrombocytopenia (platelets of 11 × 10^9^/L), lactate dehydrogenase (LDH) of 183 U/l, reticulocyte count of 1.82%, serum iron of 38 μg/dl, transferrin saturation of 11.8%, and ecchymosis with severe hemorrhagic events in the second month of gestation. Prothrombin and activated thromboplastin time were normal.

Her physical examination on admission to our hospital revealed that she was a conscious woman who was afebrile, anicteric, pale, and with a good general condition. She was oriented and cooperative and no edema was observed. Her mental status was normal. There was no spinal deformity or tenderness, no subcutaneous nodules, and no focal neurological deficits. A motor and sensory examination did not show abnormalities. Her neurological examination was normal. A medical examination was remarkable for mild petechial lesions in her lower and upper limbs. She had a blood pressure (BP) value of 140/80 mmHg. An ultrasound examination on admission showed a fetus in a transverse position without biometrics abnormality detection, and normal amniotic fluid and placenta. The fetal heart rate was normal (140 beats per minute) and there were no contractions. Her laboratory data (Table 1) revealed no other changes and renal failure was not presented. She has no history of excessive alcoholic drinking, tobacco smoking, or illicit drug usage. Her family history shows no neurological diseases and no hematologic diseases. However, her cousin had thrombocytopenia and died without a definitive diagnosis.

**Table 1:** Laboratory tests performed on the first day after admission and results

Complete blood count		Coagulation	
Erythrocytes	3.52 × 10^12^ cells/L	PT-INR	1
Hemoglobin	6.5 g/dL	aPTT	35 seconds
Hematocrit	27.7%	Fib	4.41 g/L
White blood cells	9.94 × 10^9^/L	Other tests	
Platelets	11 × 10^9^/L	AST	35 U/L
Reticulocyte count	1.92%	ALT	19 U/L
		Cr	1.1 mg/dL
		Urea	40 mg/dL
		Na	140 mEq/L
		K	3.9 mEq/L
		Total bilirubin	1.0 mg/dL
		Uric acid	7.3 mg/dL
		Lactate dehydrogenase	183 U/L
		Ferritin blood test	148 ng/Ml
		Transferrin saturation	11.8%
		Serum iron	38 μg/dL
		B12 vitamin	668.4 pg/mL

*ALT* alanine aminotransferase, *aPTT* activated partial thromboplastin time, *AST* aspartate aminotransferase, *Cr* creatinine, *Fib* fibrinogen, *K* potassium, *Na* sodium, *PT-INR* prothrombin time-international normalized ratio

**Fig. 1:**
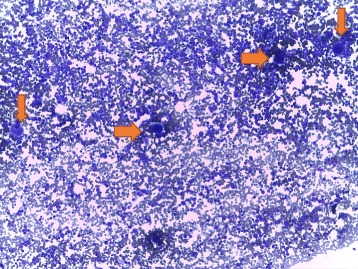
Patient’s bone marrow aspiration showing megakaryocyte hyperplasia (*orange arrows*)

**Fig. 2:**
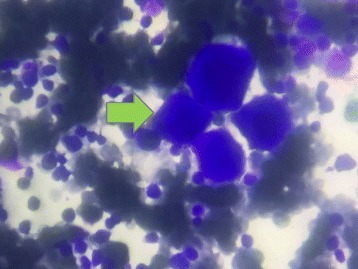
Patient’s bone marrow aspiration showing megakaryocytes (*green arrow*)

**Fig. 3:**
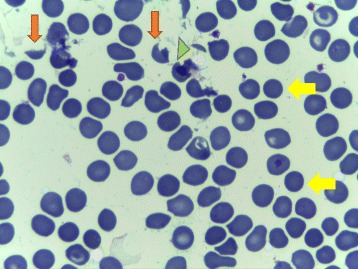
Patient’s peripheral blood smear showing microangiopathic hemolytic anemia. The smear presents schistocytes: helmet cells (*orange arrows*) and microspherocytes (*yellow arrows*). Fragmented red cells are also seen (*green arrowhead*). The platelet number is reduced

**Fig. 4:**
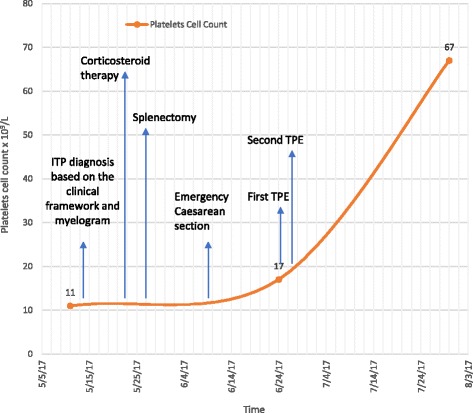
Time course of clinical symptoms, treatment, and examinations. Change in platelets before and after therapeutic plasma exchange. *TPE* therapeutic plasma exchange

During clinical investigation in the HEMOPE (Fundação de Hematologia e Hemoterapia de Pernambuco), our patient obtained an initial diagnosis suggestive of ITP based on the clinical framework, absence of schizocytes (triangular, helmet red blood cells) in peripheral blood, and nonspecific myelogram (Fig. 1). Her bone marrow was hypercellular for erythroid (27% cellularity) and had granulocytic (61% cellularity) and megakaryocytic sectors, which showed some signs of atypicality and the presence of decreased platelet genesis (Fig. 2). Steroid therapy with prednisone (60 mg per day) was started, but without adequate response, treatment with immunoglobulin was performed. Our patient remained with platelets of 17 × 10^9^/L with severe hemorrhagic events (gingivorrhagia, epistaxis), and splenectomy was indicated. One week after this surgery, she showed high BP, headache, hepatic enzyme alterations, and LDH of 1675 U/l evolving to cesarean delivery with 27 weeks' gestation in another emergency hospital due to the suggestive framework of hemolysis with elevated liver enzymes and low platelet count (HELLP) syndrome raised by the Medical Obstetrics Board. The baby was born healthy and with no clinical complications so far. Due to the persistence of MAHA, thrombocytopenia in the puerperium, and the presence of schizocytes in a new peripheral blood sample (Fig. 3), the hypothesis of TTP was proposed, since the persistence of the clinical framework did not support the hypothesis of HELLP syndrome. Plasmapheresis was indicated. After two sessions, there was an increase in platelet counts of 17 × 10^9^/L to 67 × 10^9^/L. Her serum level of LDH (210 U/l) was normalized in 1 week after plasmapheresis. Figure 4 shows the evolution of platelet counts before and after plasmapheresis. The results of ADAMTS 13 enzyme activity < 5% were received with no inhibitor present, with confirmed diagnosis of congenital TTP and not acquired TTP or other differential diagnosis. The clinical course of clinical symptoms, treatment, and clinical findings are shown in Fig. 4.

During the following 2 months, she developed recurring monthly episodes of TTP with hypochromic anemia, thrombocytopenia, hemorrhagic events, and petechial lesions in her body that indicated initiation of a preventive fresh frozen plasma (FFP) monthly infusion (10 mL/kg per dose). She has remained free of recurrences without schizocytes in her blood count since preventive treatment was started in September 2017. Up until now, her child does not have thrombocytopenia or symptoms of the disease. He has been asymptomatic without any treatment.

## Discussion

We report here the clinical and biological findings of a congenital TTP during pregnancy in a 20-year-old woman which was misdiagnosed as ITP and had been wrongly referred to splenectomy and cesarean delivery.

TTP is primarily caused by an autoimmune mechanism. However, it is also described as a non-immune hereditary form: inherited TTP or Upshaw–Schulman syndrome (USS) is rare with only 100 cases described worldwide. Inherited TTP is caused by a mutation in the *ADAMTS13* gene (9q34), encoding ADAMTS13, a metalloprotease involved in the cleavage of ultra-large vWF multimers with a penetrance of over 90%. Early TTP in childhood and adolescence represents < 10% of all cases of TTP. Due to its rarity, many cases are underdiagnosed (approximately 30% as hemolytic-uremic syndrome, immune thrombocytopenia, Evans syndrome, or hematological malignancy). The initial episode of inherited TTP occurs during childhood or neonatal period, which may suggest that the neonatal period is a time of physiologic stress for this disease. The clinical manifestations are highly variable with mild manifestations of isolated thrombocytopenia throughout childhood in some children, and severe neonatal hyperbilirubinemia with episodes of thrombocytopenia and MAHA developing soon after birth in other patients. The severity of the disease is variable and related to specific mutations of *ADAMTS13*. There is also a greater renal dysfunction in the non-idiopathic form [1].

When associated with pregnancy, most symptomatic cases of TTP occur during the second half of pregnancy and are characterized by a high frequency of USS (33%), which justifies why our patient was not diagnosed earlier [7]. This is the main cause of attacks during pregnancy, along with a hypercoagulable state with ADAMTS13 deficiency [4].

Misdiagnoses may occur because hereditary TTP is a very rare hemolytic anemia and, along with thrombocytopenia, is attributed to other causes such as ITP, HELLP syndrome, and acquired TTP. In this case, the misdiagnosis of ITP occurred because ITP is not typically associated with microangiopathic changes on the peripheral blood smear such as schistocytes and does not cause renal or neurological abnormalities (unless caused by bleeding, which is rare) [8]. However, ITP was a wrong diagnosis because our patient did not present severe ADAMTS13 deficiency [9].

Other systemic pregnancy-associated syndromes such as HELLP may lead to a misdiagnosis of inherited TTP. HELLP syndrome (hemolytic anemia, elevated liver enzymes and low platelet count) occurs with pregnancy induced by preeclampsia or hypertensive nephropathy, and may cause multiple organ failure [10]. HELLP diagnostic criteria (low serum haptoglobin levels and elevated indirect bilirubin levels in association with elevation in liver enzymes after ruling out other causes of hemolysis and thrombocytopenia) are not useful for discriminating between hereditary TTP and HELLP because both diseases may be associated with thrombocytopenia and hemolytic anemia with microangiopathic changes on the peripheral blood smear [11]. Differentiating HELLP syndrome from TTP is occasionally possible when abnormalities persist following delivery [12], and patients with HELLP syndrome do not present severe ADAMTS13 deficiency and require the treatment of the underlying syndrome such as delivery rather than plasma infusion, which is for inherited TTP. In this case, the early detection (at second month of pregnancy) of anemia and thrombocytopenia is also unusual in HELLP syndrome, a possible sign to avoid the misdiagnosis of HELLP syndrome.

Acquired TTP is more common than inherited TTP. Both are characterized by thrombocytopenia and MAHA without other causes, and may present renal insufficiency or neurologic abnormalities. They are caused by severely deficient activity of the vWF protease ADAMTS13 (activity < 10%). However, acquired TTP is caused by an inhibitory autoantibody, and this usually can be detected at the same time as ADAMTS13 measurement [13].

In the case of USS, TTP occurs as early as the first pregnancy, while the acquired form may occur at any gestation period. The fetal loss rate is high (40%) due to erroneous and late diagnoses, and fetal extraction does not improve TTP symptoms. Clinical pain (fever, thrombocytopenia, MAHA, renal failure, and neurologic symptoms) is observed in < 10% of patients, and the most frequent signs are MAHA and severe thrombocytopenia (usually < 30,000). In congenital TTP, plasma infusion restores ADAMTS13 levels and improves symptoms. Immunosuppressive therapy is unnecessary [4].

The possibility of hereditary TTP diagnosis should be considered in a patient who presents MAHA and thrombocytopenia. A laboratory study reported schistocytes on peripheral blood smears, low platelet counts, and ADAMTS13 deficiency (<10% normal values) in the absence of anti-ADAMTS13 antibodies, suggesting a diagnosis of congenital TTP [14].

Acute episodes of congenital TTP may be treated by plasma infusion (10–15 ml/kg per day), but exchange transfusion is usually required for newborns [15]. Some patients may require chronic treatment with periodic therapeutic plasma exchange, while others can only be treated when their condition worsens. At the time of the onset of acute episodes, the physician should monitor clinical impacts to minimize the patient’s risks. Patients with a chronic relapsing disease course may be considered for prophylactic plasma therapy. Regular plasma infusions to maintain ADAMTS13 activity levels around 15% are required during pregnancy [16].

In the absence of treatment, TTP is a rapidly fatal disease (mortality rate > 90%). The introduction of therapeutic plasma exchange and plasma infusion has led to a decrease in the mortality rate to around 15% [17].

Our reported case of a pregnant woman with inherited TTP is unusual because this case presents atypical clinical features of hereditary ADAMTS13 deficiency. Clear signs of hemolysis, renal failure, or neurological symptoms are not present which can indicate that clinical manifestations of inherited TTP are extremely variable. Inherited TTP is not a widely known mimicker of ITP. Although repeated peripheral blood is recommended to exclude inherited TTP in patients with recurrent thrombocytopenia, our patient did not present schistocytes in repeated peripheral blood.

## Conclusions

Congenital TTP should be included in differential diagnoses. It is essential to determine the ADAMTS13 activity in patients with thrombocytopenia with an unknown etiology. Elucidating the correct and early detection of ADAMTS13 deficiency is relevant for the patient's prognosis, reducing morbidity and mortality. The correct diagnosis allows appropriate treatment, which is an important fact to prevent severe sequels and to avoid useless therapies such as steroid therapy.

## Abbreviations

BP: Blood pressure
FFP: Fresh frozen plasma
HELLP: Hemolysis with elevated liver enzymes and low platelet count
HEMOPE: Fundação de Hematologia e Hemoterapia de Pernambuco
ITP: Immune thrombocytopenic purpura
LDH: Lactate dehydrogenase
MAHA: Microangiopathic hemolytic anemia
TTP: Thrombotic thrombocytopenic purpura
USS: Upshaw–Schulman syndrome
vWF: von Willebrand factor
